# New Diterpenoids from *Clerodendranthus spicatus*

**DOI:** 10.1007/s13659-017-0128-8

**Published:** 2017-05-03

**Authors:** Ya-Mei Li, Bing Xiang, Xiao-Zheng Li, Yong-Ming Yan, Yong-Xian Cheng

**Affiliations:** 1 Yunnan University of Traditional Chinese Medicine, Kunming, 650504 People’s Republic of China; 2 State Key Laboratory of Phytochemistry and Plant Resources in West China, Kunming Institute of Botany, Chinese Academy of Sciences, Kunming, 650201 People’s Republic of China

**Keywords:** *Clerodendranthus spicatus*, Diterpenoid, Neoorthosiphonones B and C

## Abstract

Two new diterpenoids, neoorthosiphonones B and C (**1** and **2**), and one known diterpenoid, were isolated from the aerial parts of *Clerodendranthus spicatus*. Their structures including absolute configurations were determined by comprehensive spectroscopic analyses and X-ray crystallographic methods. No compound was found to inhibit fibronectin production at the concentration of 20 μM.

**Electronic supplementary material:**

The online version of this article (doi:10.1007/s13659-017-0128-8) contains supplementary material, which is available to authorized users.

## Introduction

**Fig. 1:**
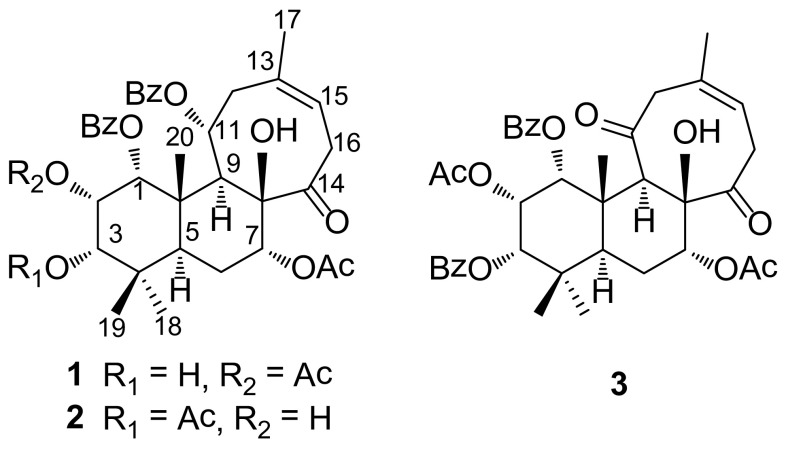
The structures of compounds **1**–**3**

*Clerodendranthus spicatus* (Thunb.) is a traditional medicine of Dai nationality of Yunnan Province, China, which has been used for the treatment of acute and chronic nephritis, diabetes, urocystitis, urinary lithiasis, rheumatism, and renal diseases [1]. Modern pharmacological studies showed that *C. spicatus* has a wide range of effects including anti-inflammation, diuresis, antibiosis, renal function improvement, anti-hyperglycemia, anti-gout, pressure release, anti-tumor and immunity improvement [2]. Previous chemical investigations on *C. spicatus* mainly focused on flavonoids [3], diterpenoids [4–6] phenolics, polyphenolic acids [7–10] triterpenes, and volatile oil [1]. Our recent study on *C. spicatus* disclosed eighteen compounds including lignans, flavonoids, and other type of compounds [11]. As a continuation of our work, our study focusing on diterpenoids from *C. spicatus* led to the isolation of two new diterpenoids, named neoorthosiphonones B and C, and one known compound neoorthosiphonone A (Fig. 1) [12]. In this paper, we describe their isolation and structure elucidation. In addition, biological activities of these diterpenoids towards kidney protection were carried out.

## Results and Discussion

**Fig. 2:**
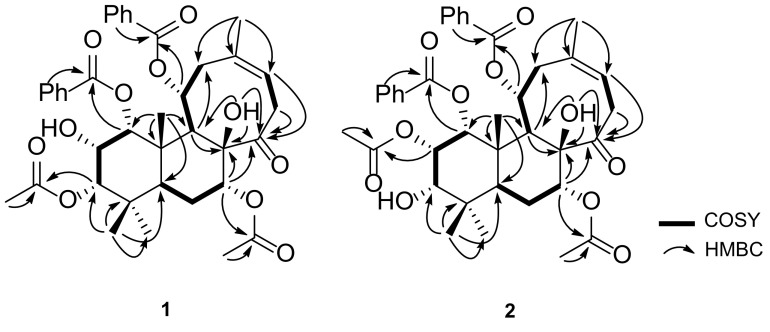
^1^H–^1^H COSY and HMBC correlations of compounds **1** and **2**

**Fig. 3:**
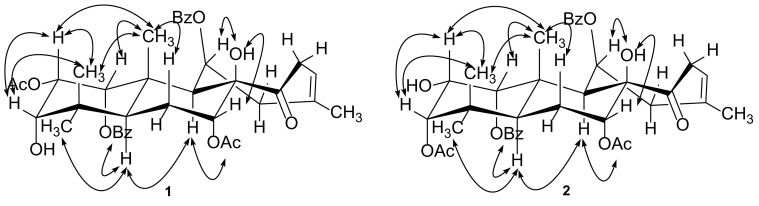
ROESY correlations of compounds **1** and **2**

**Fig. 4:**
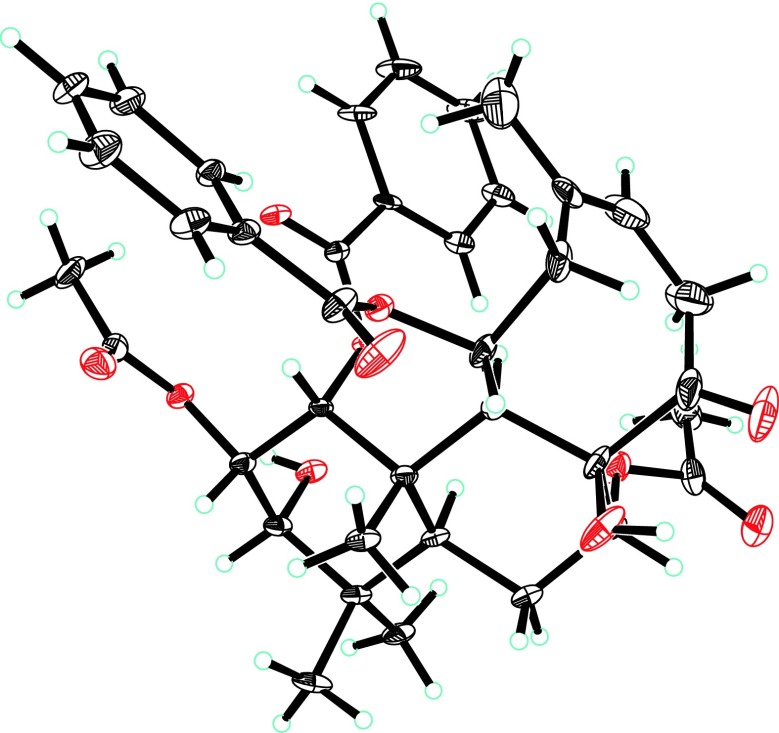
The X-ray structure of **1**

Neoorthosiphonone B (**1**) was found to have the molecular formula C_38_H_44_O_11_ (17° of unsaturation) derived by analysis of its HRESIMS at *m/z* 699.2798 [M+Na]^+^ (calcd for C_38_H_44_O_11_Na, 699.2776), ^13^C NMR, and DEPT spectra. The ^1^H NMR spectrum (Table 1) of **1** contains two typical benzoyl group, an olefinic signal [*δ*_H_ 4.37 (brt, *J* = 5.0 Hz, H-15)], five oxygenated methines [*δ*_H_ 5.93 (brs, H-1), *δ*_H_ 5.23 (brt-like, *J* = 2.9 Hz, H-2), *δ*_H_ 3.57 (brs, H-3), *δ*_H_ 4.52 (brs, H-7), *δ*_H_ 5.87 (dt, *J* = 10.6, 3.0 Hz, H-11)], and six methyls [*δ*_H_ 1.39 (s, H-17), *δ*_H_ 1.08 (s, H-18), *δ*_H_ 1.01 (s, H-19), *δ*_H_ 1.26 (s, H-20), *δ*_H_ 2.01 (s, 2-OCOCH_3_), *δ*_H_ 2.20 (s, 7-OCOCH_3_)]. The ^13^C NMR and DEPT spectra demonstrate resonances for 38 carbons including six methyl, three aliphatic methylene, eighteen methine (seven aliphatic including five oxygenated, eleven olefinic/aromatic), and eleven quaternary carbons (three aliphatic including one oxygenated, three olefinic/aromatic, four ester carbonyls, one ketone). These NMR data, in consideration of chemical profiling of *C. spicatus*, prompt us to speculate that **1** is likely a diterpenoidal derivative similar to compound **3** [12]. Careful analysis of NMR data (Fig. 2) of **1** disclosed that **1** differs from **3** in two places. The ^1^H–^1^H COSY correlations of H-9/H-11 (*δ*_H_ 5.87)/H_2_-12 and HMBC correlations of H-9/C-11 (*δ*_C_ 74.2), H-11, H-2′′ or H-6′′/C-7′′ clearly indicates that a benzoyl group is positioned at C-11 in **1** rather than a ketone in **3**. In addition, **1** differs from **3** in that a benzoyl group at C-3 of **3** is absent in **1** supported by the observation of the distinct up-field shift of H-3 of **1** (∆ 1.80 ppm). The relative configuration of **1** was established mainly by ROESY data (Fig. 3). ROESY correlations of H_3_-18, H_3_-19/H-3β; H_3_-19/H-2β, H_3_-20; H_3_-20/H-1β, H-2β; 8-OH/H-7β, H-11β, H_3_-18/H-5, H-5/H-2′,6′, H-5/H-9, H-9/7-OCOCH_3_ are observed, indicating the relative configurations at C-1, C-2, C-3, C-5, C-7, C-8, C-9, and C-11. There is one double bond in the structure of **1**, whose geometry was assigned as *Z* by the ROESY correlation of H-15/H_3_-17. The absolute configuration of **1** was finally determined to be 1*R*, 2*S*, 3*S*, 5*S*, 7*R*, 8*R*, 9*S*, 10*S*, 11*S* (Fig. 4) by single-crystal X-ray diffraction with Cu Kα radiation. Consequently, the structure of **1** was characterized and named as neoorthosiphonone B.

**Table 1:** ^1^H and ^13^C NMR spectral data (600 MHz for ^1^H and 150 MHz for ^13^C in CDCl_3_) of compounds **1** and **2** (*δ* in ppm, *J* in Hz)

No.	**1**		**2**	
	*δ*_H_	*δ*_C_	*δ*_H_	*δ*_C_
1	5.93, brs	77.7	5.57, brs	81.3
2	5.23, brt-like (2.9)	67.8	4.22, m	65.9
2-OH			3.96, brd (5.0)	
3	3.57, brs	76.7	5.05, brd (2.7)	78.2
4		38.2		37.1
5	2.61, brd (12.3)	34.1	2.51, brd (13.0)	35.9
6	2.06, overlap	21.3	2.08, t-like (14.0)	21.8
	1.91, d (11.8)		1.88, brd (14.0)	
7	4.52, brs	77.6	4.54, brs	77.7
8		79.7		79.9
8-OH	3.96, s		4.03, s	
9	3.28, overlap	40.5	3.60, d (10.4)	41.0
10		43.0		43.8
11	5.87, dt (10.6, 3.0)	74.2	5.89, dt (10.4, 2.6)	74.7
12	2.06, overlap	34.5	2.14, m	34.0
13		133.2		133.2
14		216.2		216.4
15	4.37, brt (5.0)	120.4	4.79, brt (5.0)	120.2
16	3.28, overlap	44.5	3.62, overlap	44.2
	3.01, brd (16.8)		3.17, brd (15.8)	
17	1.39, s	26.3	1.33, s	26.7
18	1.08, s	29.4	0.88, s	28.1
19	1.01, s	23.1	1.07, s	23.0
20	1.26, s	14.1	1.33, s	15.0
2-OCOCH_3_		169.5		
	2.01, s	20.9		
3-OCOCH_3_				170.7
			1.62, s	20.5
7-OCOCH_3_		168.1		167.8
	2.20, s	21.2	2.22, s	21.2
1-OCOPh				
1’		130.6		130.4
2′,6′	8.25, d (7.5)	129.6	8.29, d (7.4)	129.6
3′,5′	7.53, overlap	128.4	7.56, t-like (7.7)	128.5
4′	7.68, t-like (7.5)	133.5	7.72, t-like (7.4)	133.8
7′		164.4		167.8
11-OCOPh				
1″		129.6		129.4
2″,6″	8.38, d (7.5)	130.6	8.10, d (7.4)	130.3
3″,5″	7.53, overlap	128.2	7.49, t-like (7.7)	128.6
4″	7.62, t-like (7.5)	133.2	7.61, t-like (7.4)	133.6
7″		166.5		165.7

Analysis of the HRESIMS at *m/z* 715.2515 [M+K]^+^ (calcd for C_38_H_44_O_11_K, 715.2515), ^13^C NMR, and DEPT data of neoorthosiphonone C (**2**) indicates that **2** has the same molecular formula and similar structure as does **1**. Compound **2** differs **1** only in the position of an acetyl group. The ^1^H–^1^H COSY correlations of H-1/H-2/H-3 and HMBC correlations of H-18, H-19/C-3 (*δ*_C_ 78.2), 3-OCOCH_3_ (*δ*_H_ 1.62)/3-OCO (*δ*_C_ 170.7), and H-3/3-OCOCH_3_ observed in **2** indicate that **2** is a 3-acetyl positioner of **1**. To assign the relative configuration of **2**, a ROESY experiment was carried out. The ROESY spectrum shows correlations of H_3_-18, H_3_-19/H-3β; H_3_-19/H-2β, H_3_-20; H_3_-20/H-1β, H-2β; 8-OH/H-7β, H-11β, H_3_-18/H-5, H-5/H-2′,6′, H-5/H-9, H-9/7-OCOCH_3_ (Fig. 3), unambiguously assigning the relative configuration of **2**. In the same manner as that of **1**, the double bond between C-13 and C-15 was deduced to be *Z*-form due to ROESY correlation of H-15/H_3_-17. As a result, the structure of **2** was identified and named as neoorthosiphonone C.

So far, diterpenoids with several frameworks have been characterized from the title species [1]. Compounds **1** and **2** are characteristic of the presence of a 6/6/8 ring system which makes it unusual despite that neoorthosiphonone A as the only one analogue has been identified from *C. spicatus* [12].

The known compound neoorthosiphonone A (**3**) was isolated and identified by comparing its spectroscopic data with those previously reported [12].

Considering that *C. spicatus* is commonly used for renal diseases, in this study, compounds **1**–**3** were evaluated for their roles in renal protection by targeting fibronectin production in TGF-β1-induced rat kidney tubular epithelial cells using the method reported previously [13]. However, no compound was found to inhibit fibronectin production at the concentration of 20 μM. Whether these compounds are active towards the other kidney associated targets needs further investigation.

## Experimental

### General Experimental Procedures

Optical rotations were performed on a JASCO P-1020 digital polarimeter. UV spectra were obtained on a Shimadzu UV-2401PC spectrometer. NMR spectra were recorded on a Bruker Avance III 600 MHz spectrometer, with TMS as an internal standard. ESIMS and HRESIMS were measured on an API QSTAR Pulsar 1 spectrometer. C-18 silica gel (40–60 μm; Daiso Co., Japan), MCI gel CHP 20P (75–150 μm, Mitsubishi Chemical Industries, Tokyo, Japan), Sephadex LH-20 (Amersham Pharmacia, Uppsala, Sweden), and silica gel (200-300 mesh; Qingdao Marine Chemical Inc., PR China) were used for column chromatography. Silica gel GF_254_ (Qingdao Marine Chemical Inc., People’s Republic of China) was used for preparative TLC. Semi-preparative HPLC was carried out using an Agilent 1200 liquid chromatograph with a YMC-Pack ODS-A column (250 mm × 10 mm, i.d., 5 μm).

### Plant Material

*Clerodendranthus spicatus* was purchased from Kunming Zhonghao Luoshiwan Corporation of Chinese Materia Medica (Yunnan) People’s Republic of China, in September 2015. The material was identified by Mr. Bing Qiu at the Yunnan Institute of Materia Medica, and a voucher specimen (CHYX-0595) was deposited at the State Key Laboratory of Phytochemistry and Plant Resources in West China, Kunming Institute of Botany, Chinese Academy of Sciences, People’s Republic of China.

### Extraction and Isolation

The air-dried powders of *C. spicatus* (15 kg) was extracted under reflux using 70% EtOH (3 × 60 L × 2 h) to give a crude extract, which was suspended in water followed by successive extraction with petroleum ether and EtOAc. The EtOAc soluble extract (1.2 kg) was divided into seven parts, Fr. A–Fr. G, by using silica gel column chromatography eluted with a gradient of CHCl_3_/MeOH (1:0–0:1). Fr. B (230 g) was divided into eight portions (B1–B8) by MCI gel CHP 20P eluted with gradient aqueous MeOH (40–100%). Fr. B8 (6.3 g) was subjected to Sephadex LH-20 (MeOH) and followed by RP-18 column chromatography (MeOH/H_2_O, 60–100%) to provide six portions (B8-1–B8-6), Fr. B8-4 (400 mg) was subjected to Sephadex LH-20 (MeOH) followed by semi-preparative HPLC (MeOH/H_2_O, 70%) to yield compounds **1** (15.5 mg), **2** (2.0 mg), and **3** (10.0 mg).

#### Neoorthosiphonone B (**1**)

White solid; [α]_D_^26^ – 72.9 (*c* 0.53, MeOH); UV (MeOH) λ_max_ (log*ε*) 274 (3.29), 230 (4.34), 201 (4.25) nm; ESIMS *m/z* 699 [M+Na]^+^; HRESIMS *m/z* 699.2798 [M+Na]^+^ (calcd for C_38_H_44_O_11_Na, 699.2776); ^1^H and ^13^C NMR data, see Table 1.

#### Crystal Data for Neoorthosiphonone B (**1**)

C_38_H_44_O_11_, *M* = 676.73, *a* = 10.6141(5) Å, *b* = 14.6412(7) Å, *c* = 10.9554(6) Å, *α* = 90°, *β* = 97.648(2)°, *γ* = 90°, *V* = 1687.36(15) Å^3^, *T* = 100(2) K, space group *P*21, *Z* = 2, *μ*(CuKα) = 0.805 mm^−1^, 11874 reflections measured, 5296 independent reflections (*R*_*int*_ = 0.0585). The final *R*_*1*_ values were 0.0814 (*I* > 2*σ*(*I*)). The final *wR*(*F* ^2^) values were 0.2258 (*I* > 2*σ*(*I*)). The final *R*_1_ values were 0.0843 (all data). The final *wR*(*F* ^2^) values were 0.2314 (all data). The goodness of fit on *F* ^2^ was 1.035. Flack parameter = 0.14(11). Crystallographic data center (deposition number: CCDC 1534910).

#### Neoorthosiphonone C (**2**)

White solid; [α]_D_^26^ – 61.4 (*c* 0.53, MeOH); UV (MeOH) λ_max_ (log*ε*) 273 (3.24), 230 (4.36), 202 (4.27) nm; ESIMS *m/z* 699 [M+Na]^+^; HRESIMS *m/z* 715.2515 [M+K]^+^ (calcd for C_38_H_44_O_11_K, 715.2515); ^1^H and ^13^C NMR data, see Table 1.

### Bioassay

All compounds were evaluated for their effects in renal protection as previously described methods [13].

## Electronic supplementary material

Below is the link to the electronic supplementary material.

Supplementary material 1 (DOCX 3551 kb)
