# Supplementary material for: New Diterpenoids from Clerodendranthus spicatus
Source: Nat Prod Bioprospect. 2017 May 3;7(3):263–7. doi: 10.1007/s13659-017-0128-8 (PMC5481273; doi:10.1007/s13659-017-0128-8)
Supplement: Supplementary file 1 — Supplementary material 1 (DOCX 3551 kb) [file 13659_2017_128_MOESM1_ESM.docx]

**Supplementary Information**

**Novel diterpenoids from *Clerodendranthus spicatus***

Ya-Mei Li ^a,b^, Bing Xiang ^a^, Xiao-Zheng Li ^a,b^, Yong-Ming Yan ^a,*^, Yong-Xian Cheng ^a,b,*^

^a^ *State Key Laboratory of Phytochemistry and Plant Resources in West China, Kunming Institute of Botany, Chinese Academy of Sciences, Kunming 650201, China*

^b^ *Yunnan University of Traditional Chinese Medicine, Kunming 650504, People’s Republic of China*

*Corresponding authors. Tel./fax: +86 871 65223048. E-mail: yxcheng@mail.kib.ac.cn (Y.-X. Cheng); yanym@mail.kib.ac.cn (Y.-M. Yan).

**Content**

Figure S1. ^1^H NMR spectrum of **1** in CDCl_3_

Figure S2. ^13^C NMR and DEPT spectra of **1** in CDCl_3_

Figure S3. ^1^H-^1^H COSY spectrum of **1** in CDCl_3_

Figure S4. HSQC spectrum of **1** in CDCl_3_

Figure S5. HMBC spectrum of **1** in CDCl_3_

Figure S6. ROESY spectrum of **1** in CDCl_3_

Figure S7. HRESIMS of **1**

Figure S8. ^1^H NMR spectrum of **2** in CDCl_3_

Figure S9. ^13^C NMR and DEPT spectra of **2** in CDCl_3_

Figure S10. ^1^H-^1^H COSY spectrum of **2** in CDCl_3_

Figure S11. HSQC spectrum of **2** in CDCl_3_

Figure S12. HMBC spectrum of **2** in CDCl_3_

Figure S13. ROESY spectrum of **2** in CDCl_3_

Figure S14. HRESIMS of **2**


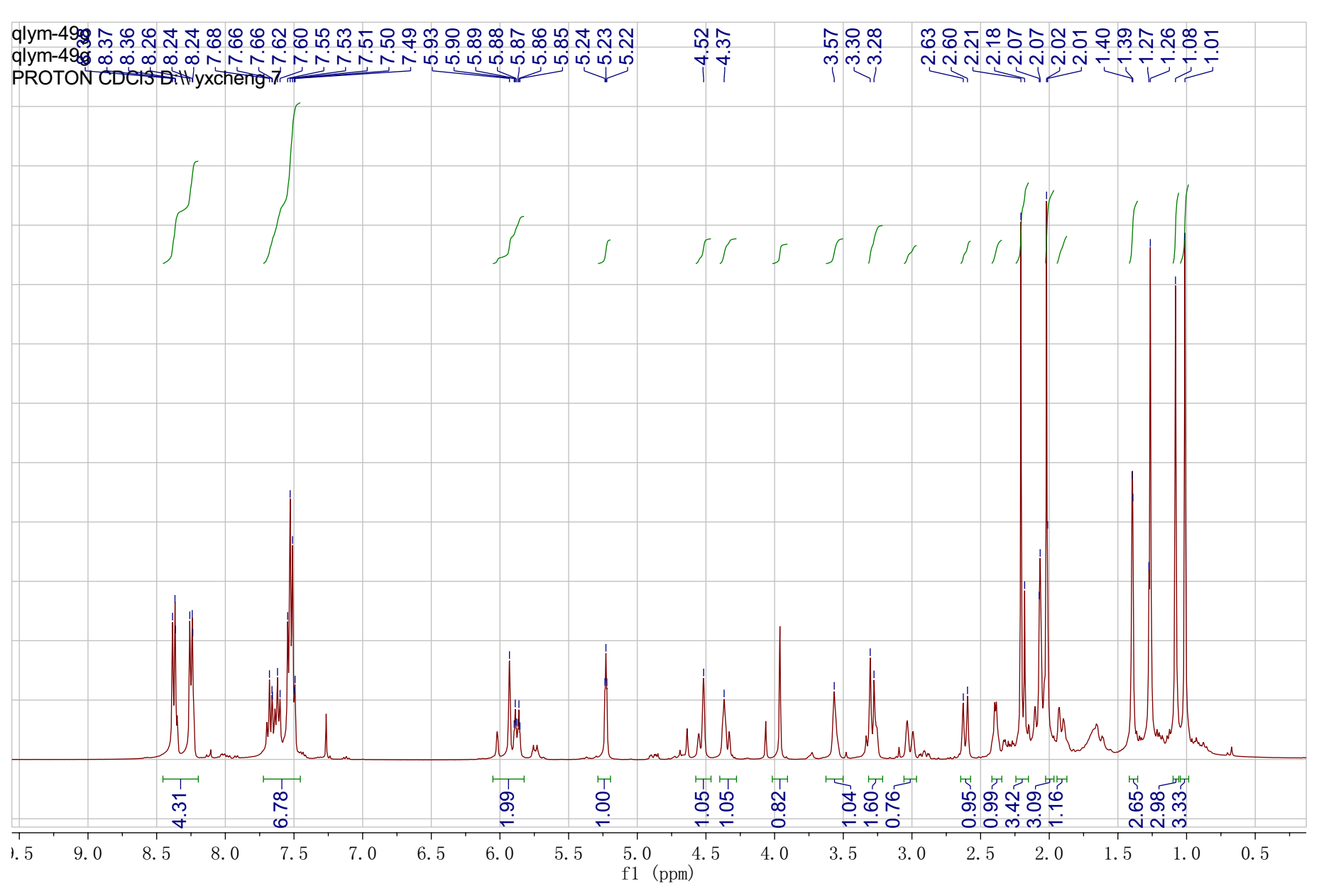


Figure S1. ^1^H NMR spectrum of **1** in CDCl_3_


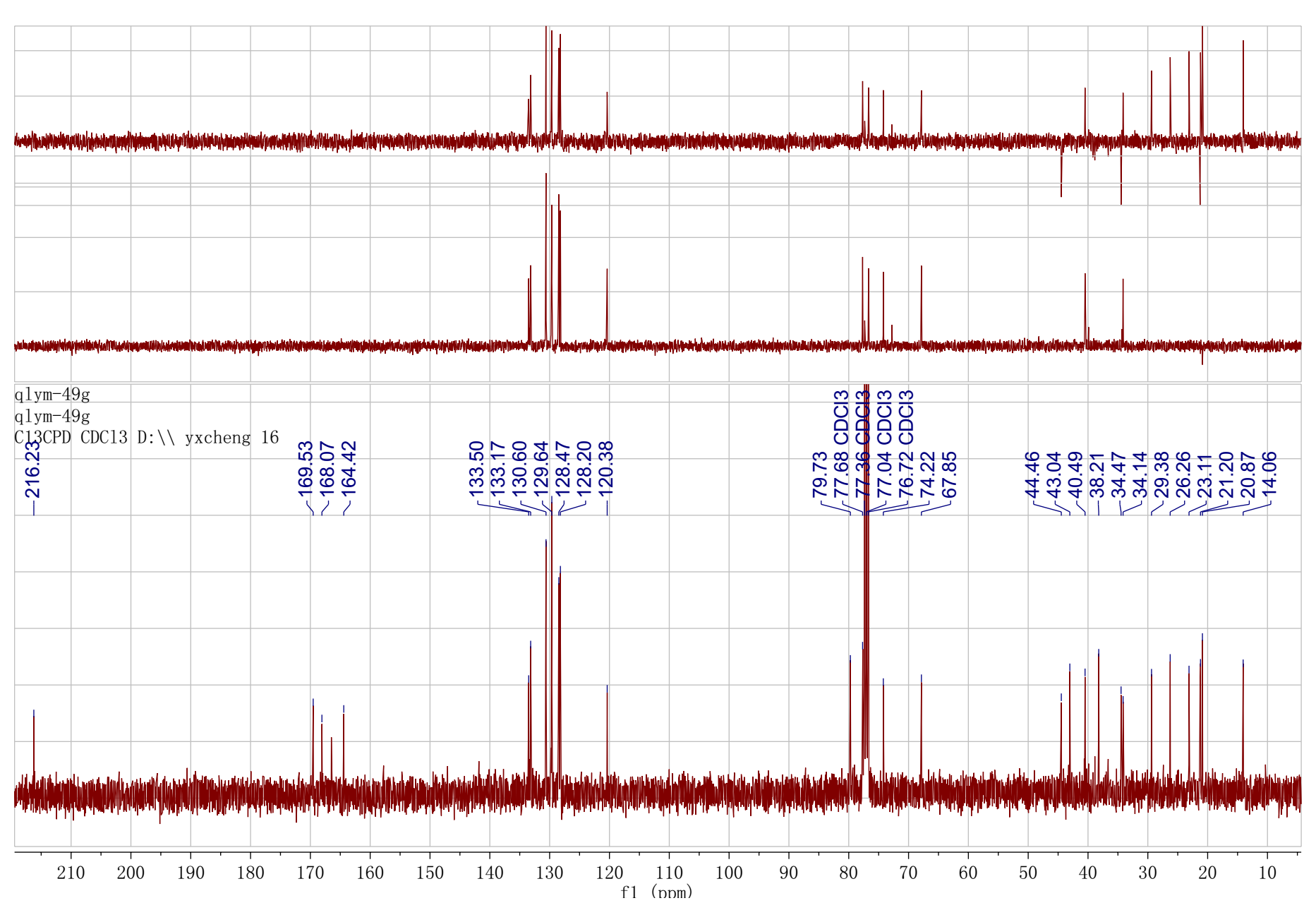


Figure S2. ^13^C NMR and DEPT spectra of **1** in CDCl_3_


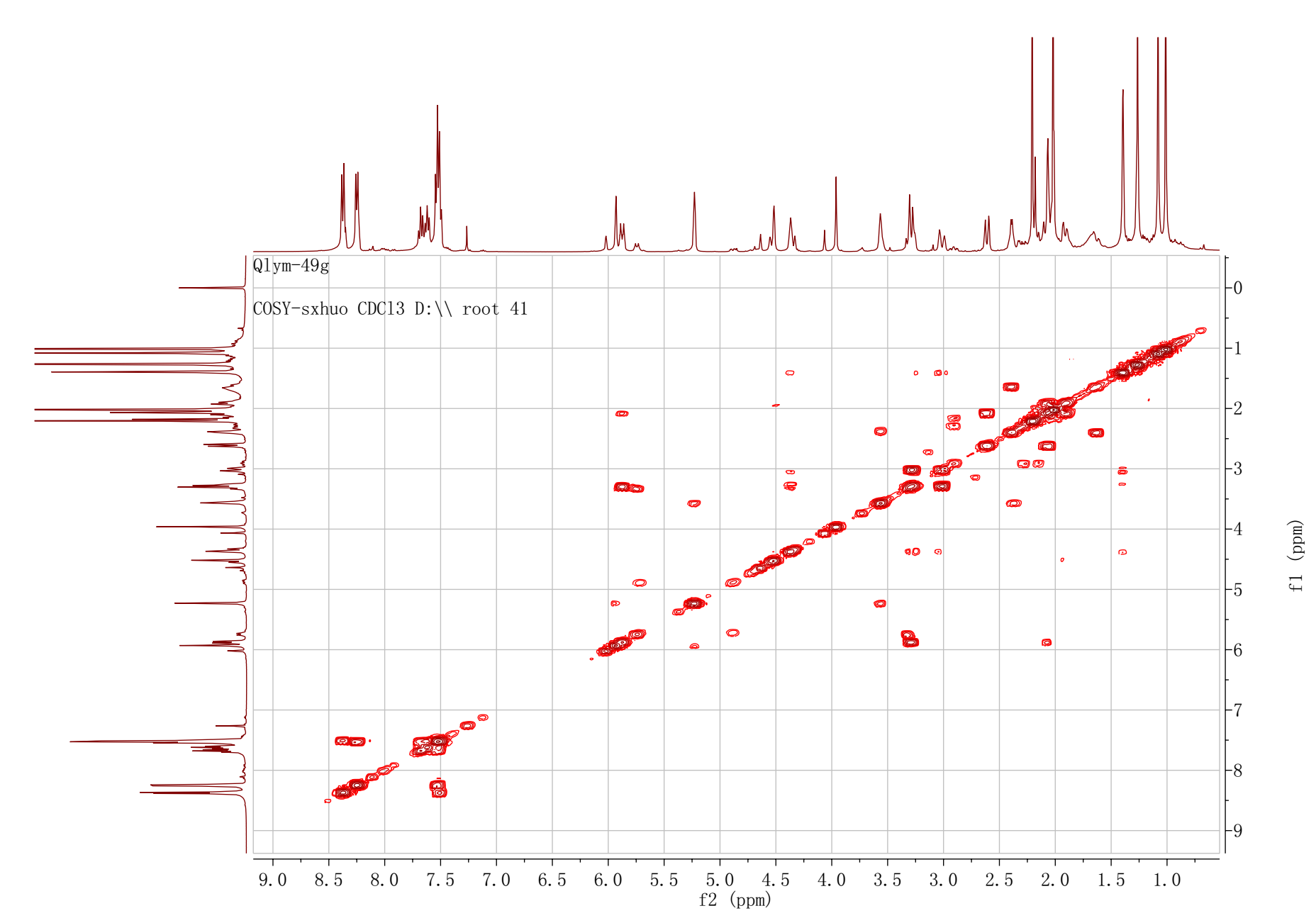


Figure S3. ^1^H-^1^H COSY spectrum of **1** in CDCl_3_


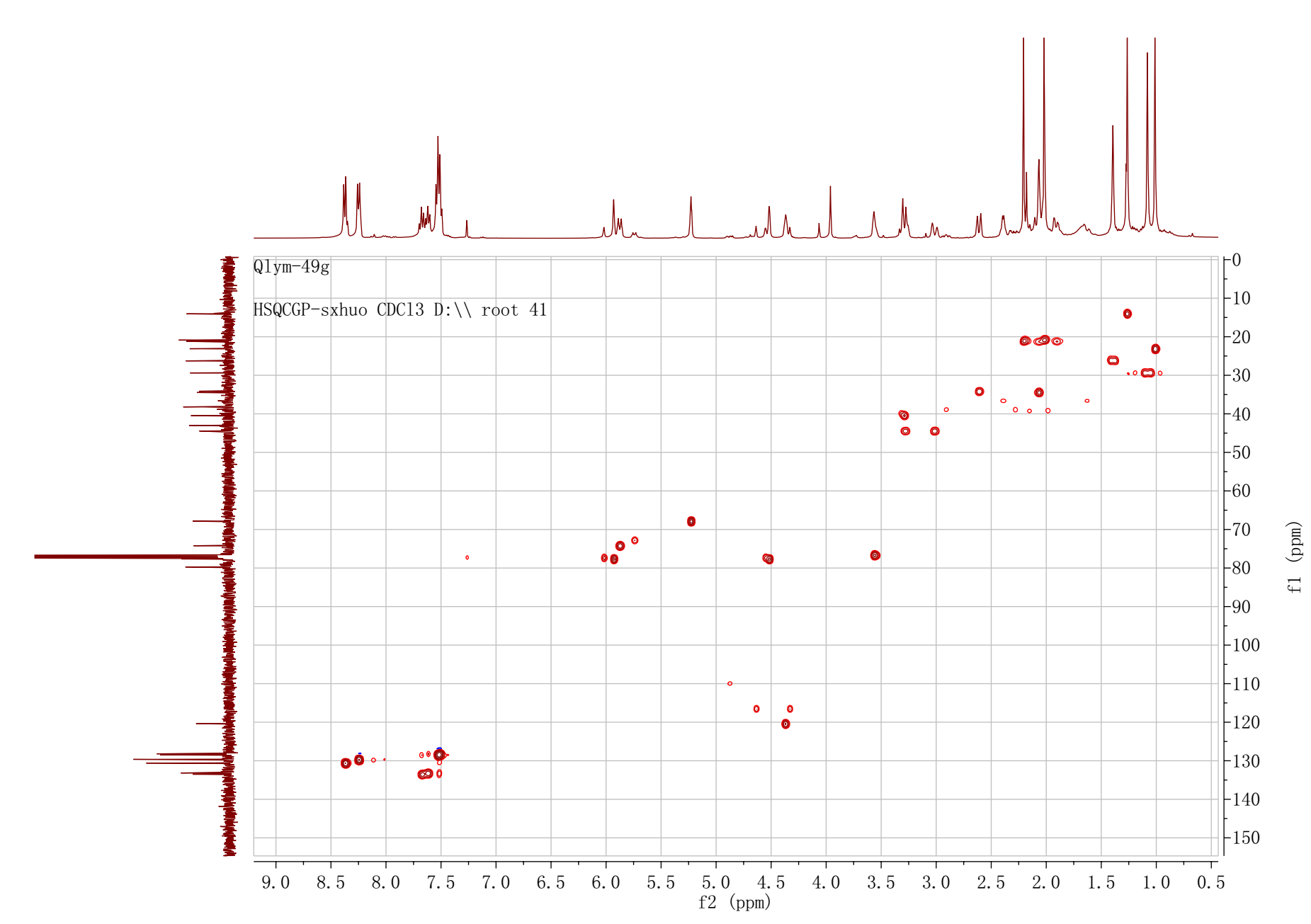


Figure S4. HSQC spectrum of **1** in CDCl_3_


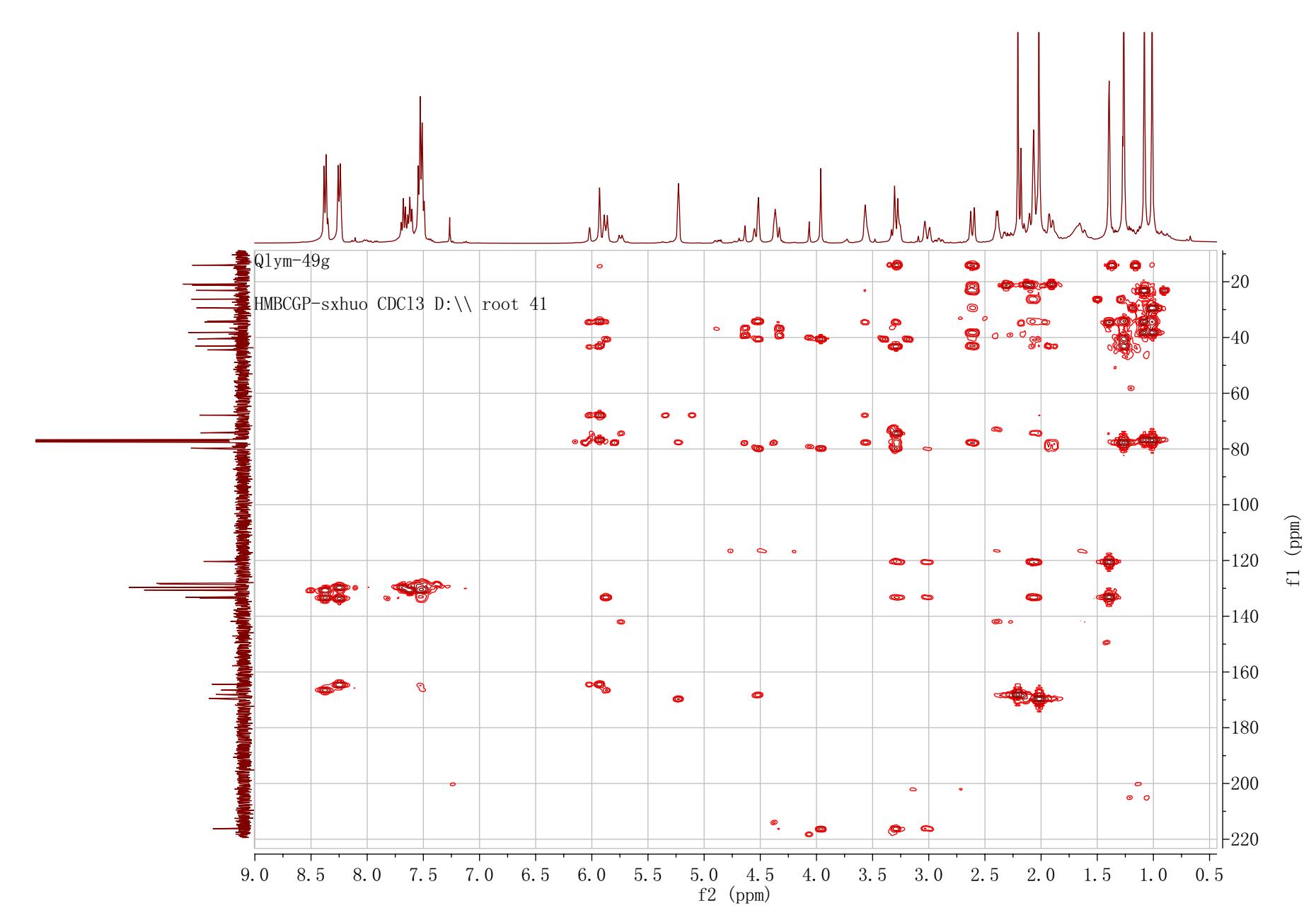


Figure S5. HMBC spectrum of **1** in CDCl_3_


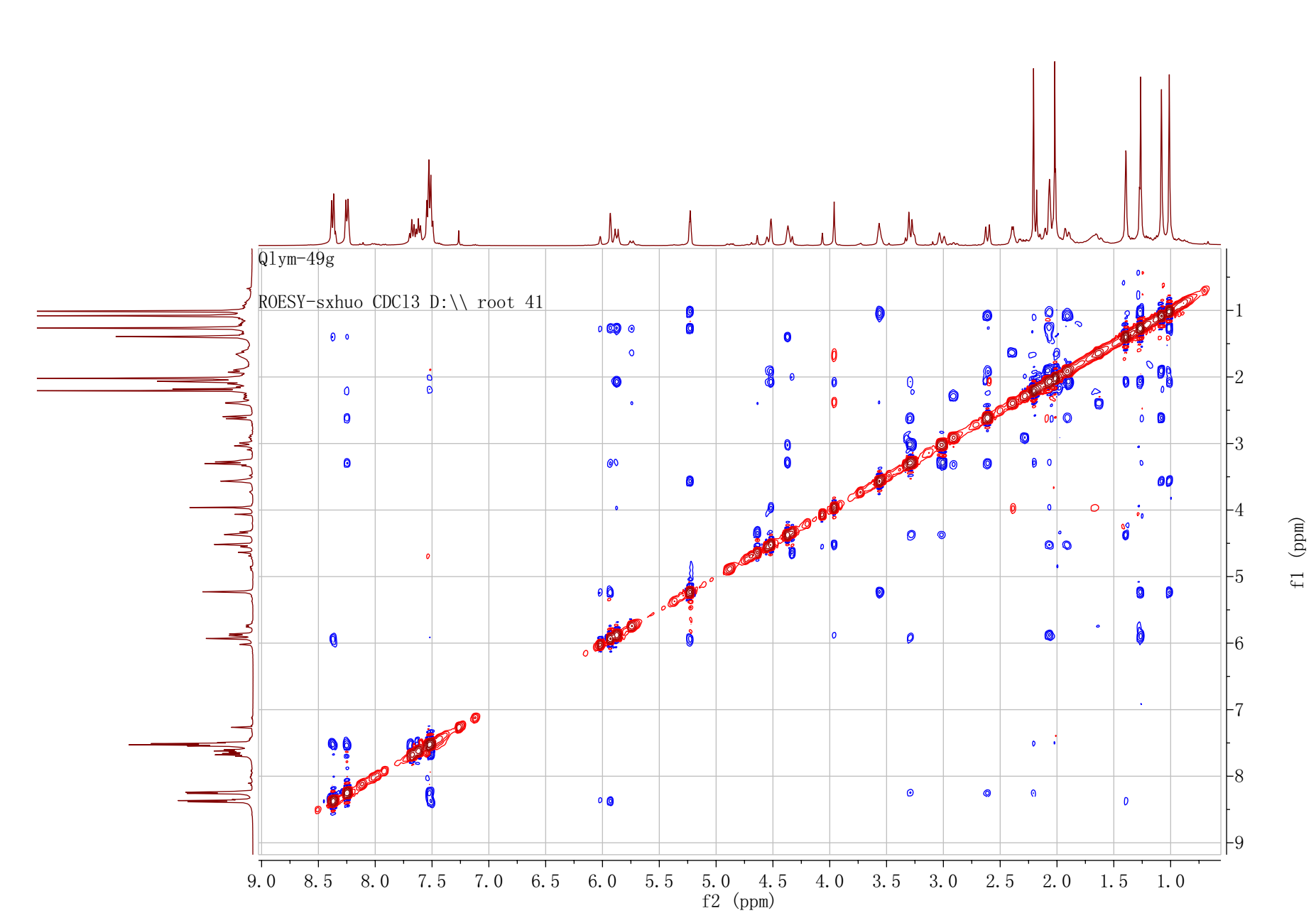


Figure S6. ROESY spectrum of **1** in CDCl_3_


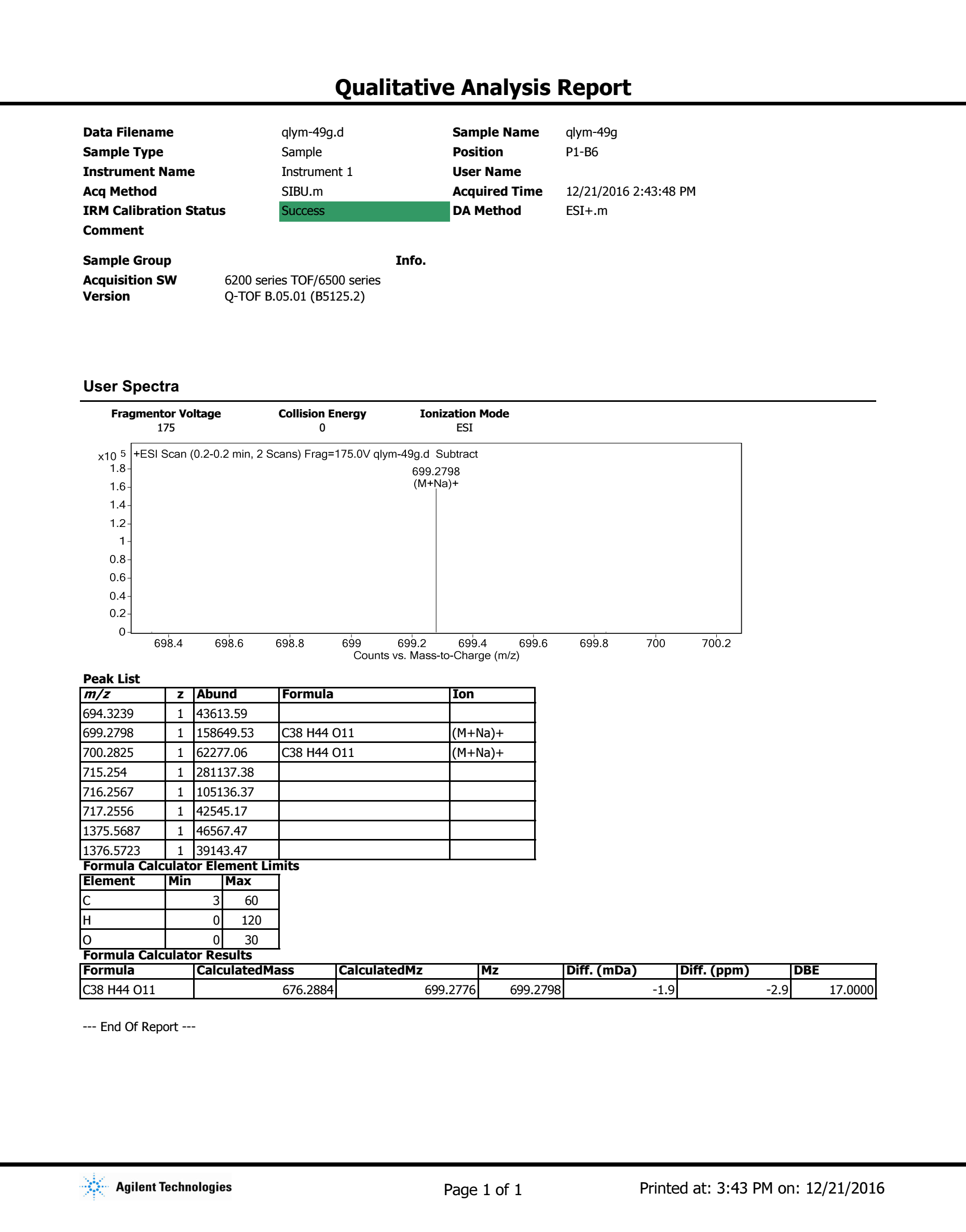


Figure S7. HRESIMS of **1**


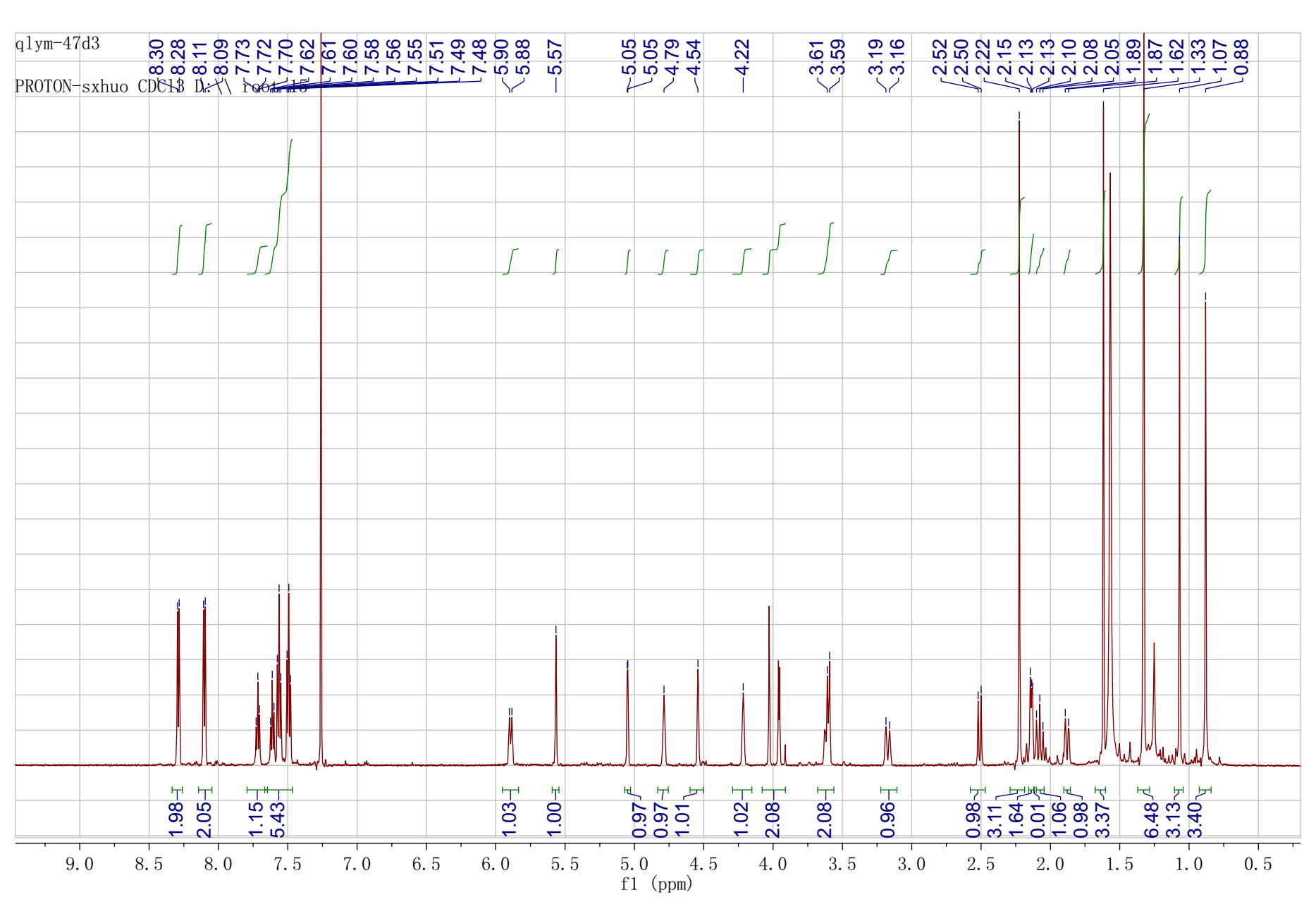


Figure S8. ^1^H NMR spectrum of **2** in CDCl_3_


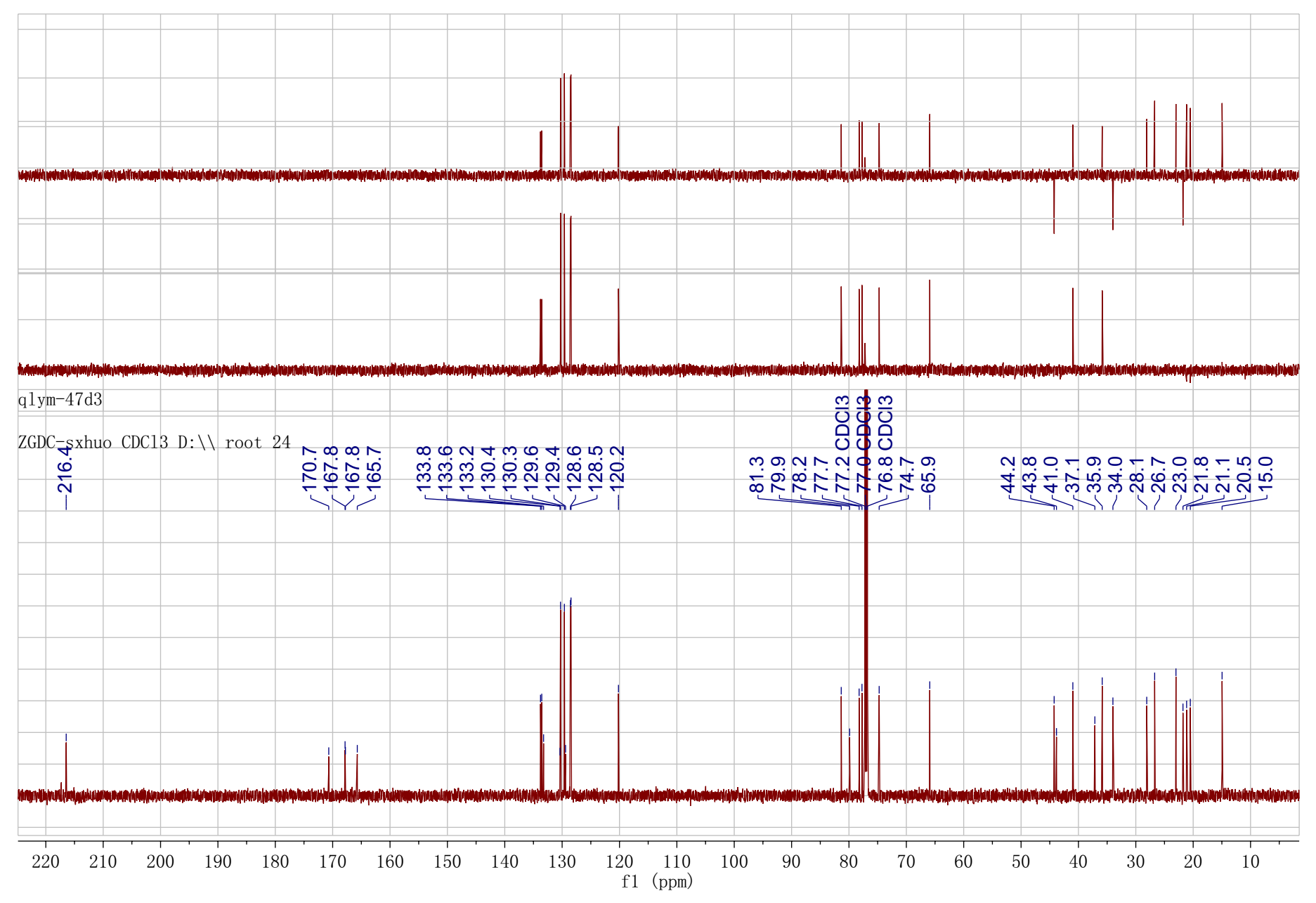


Figure S9. ^13^C NMR and DEPT spectra of **2** in CDCl_3_


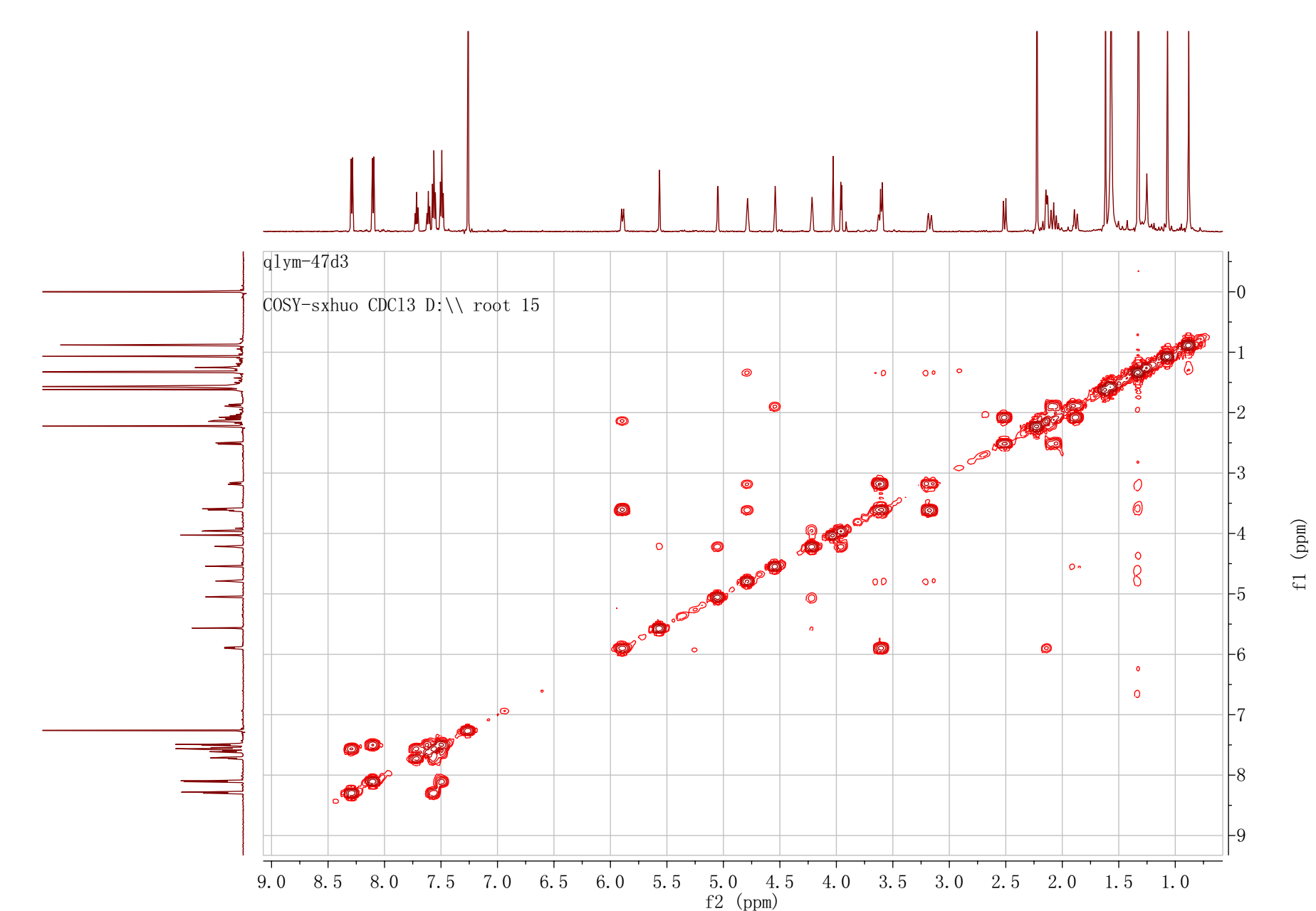


Figure S10. ^1^H-^1^H COSY spectrum of **2** in CDCl_3_


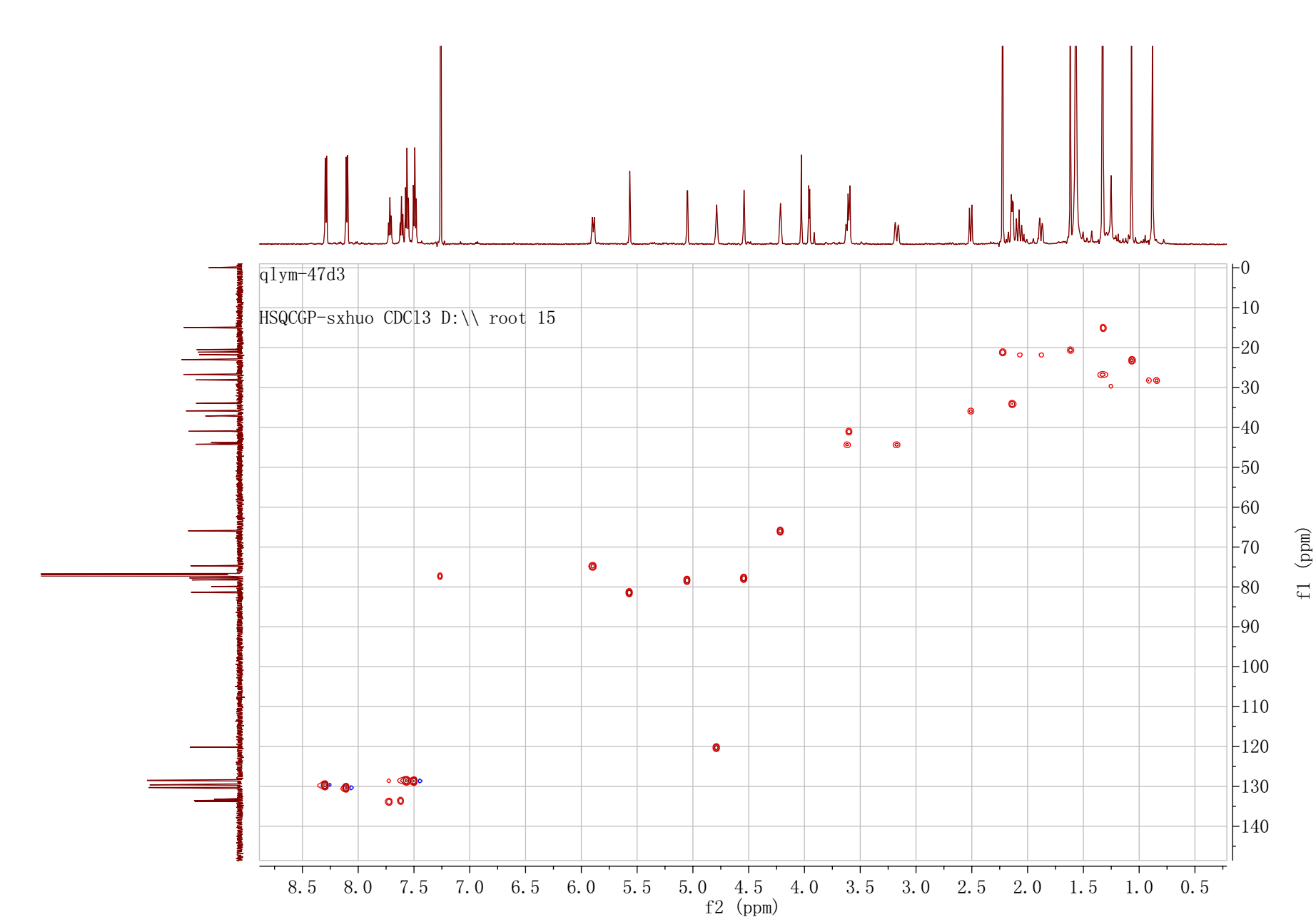


Figure S11. HSQC spectrum of **2** in CDCl_3_


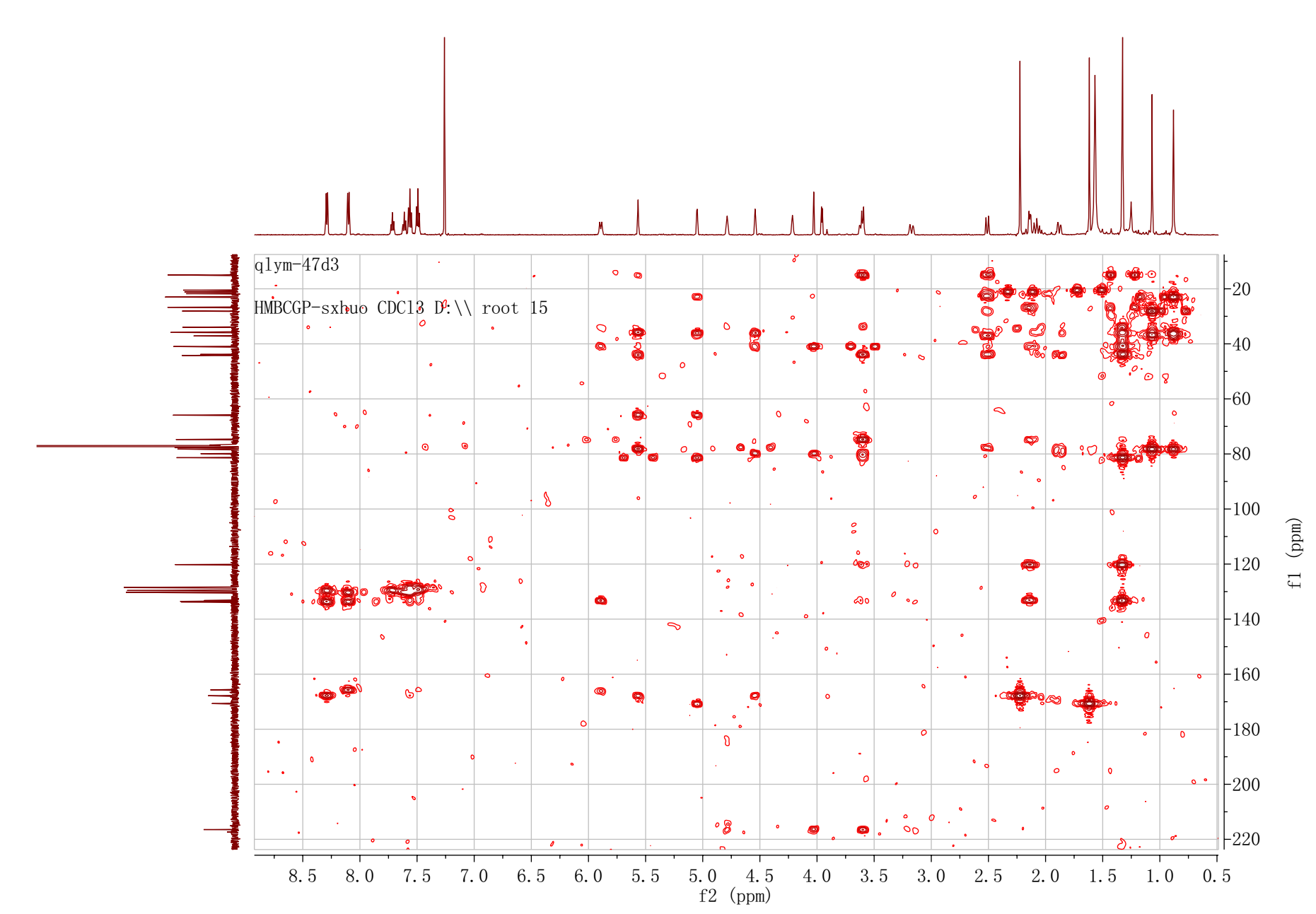


Figure S12. HMBC spectrum of **2** in CDCl_3_


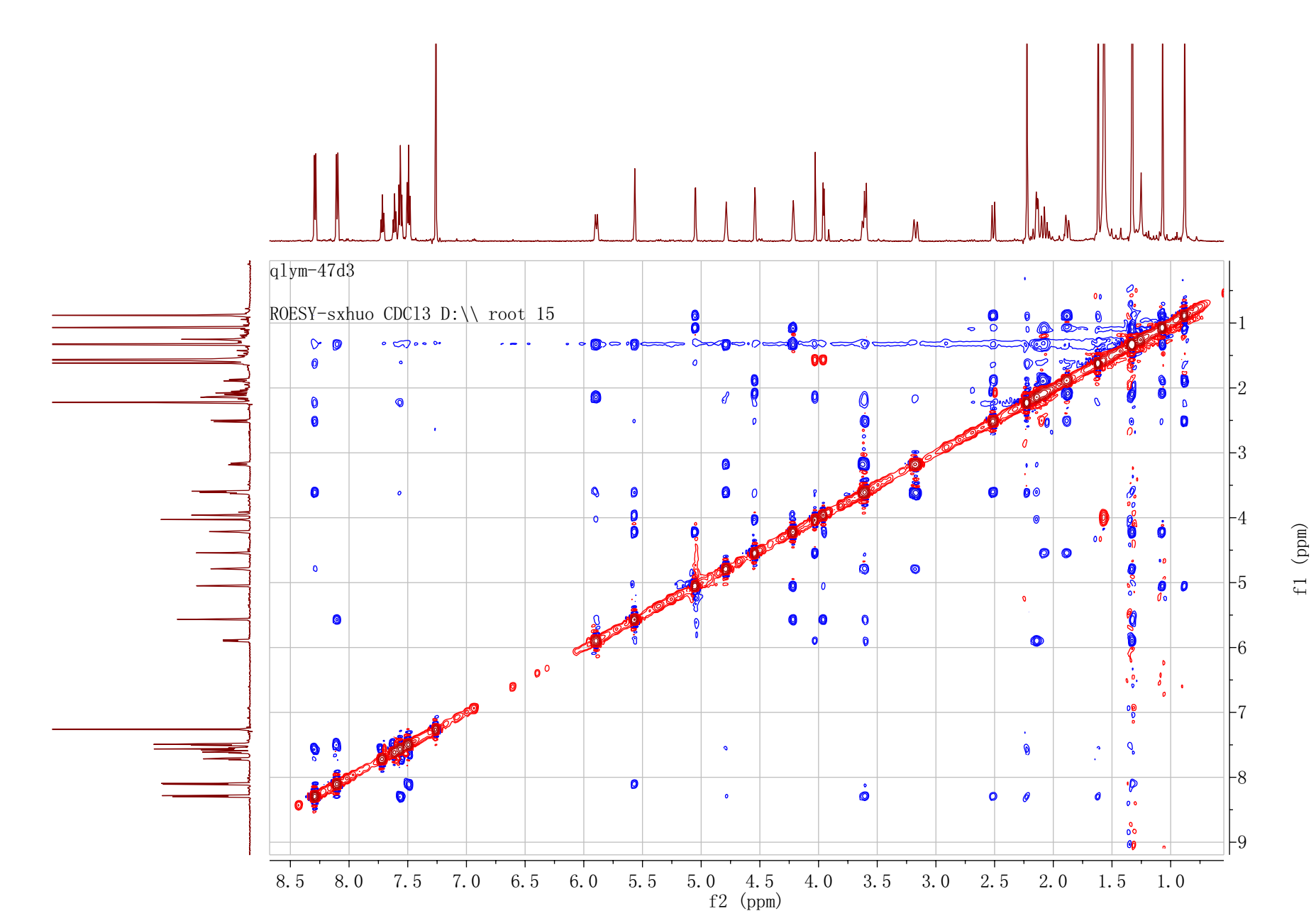


Figure S13. ROESY spectrum of **2** in CDCl_3_


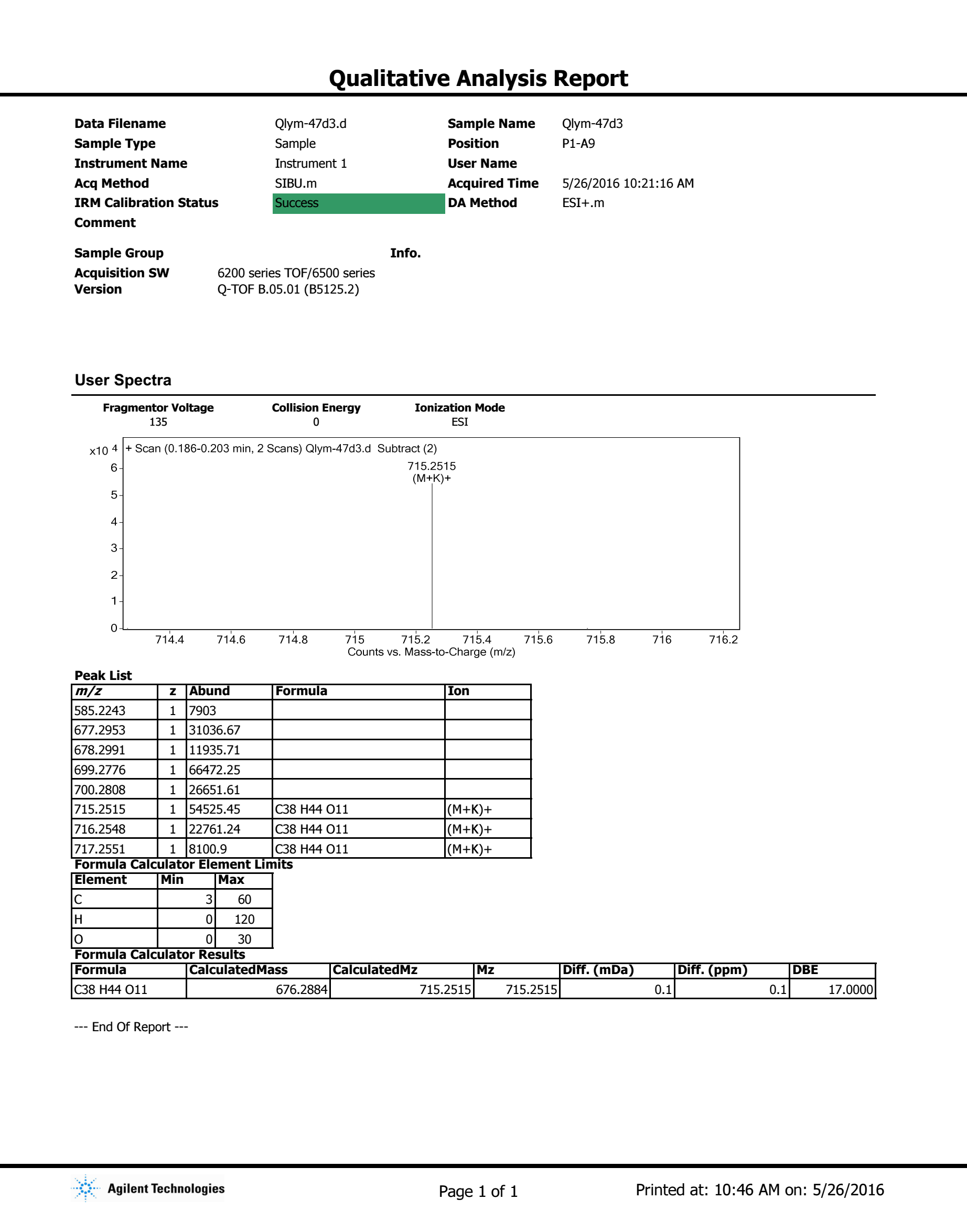


Figure S14. HRESIMS of **2**
